# Supplementary figures and images for: Deep mutational scans for ACE2 binding, RBD expression, and antibody escape in the SARS-CoV-2 Omicron BA.1 and BA.2 receptor-binding domains
Source: PLoS Pathog. 2022 Nov 18;18(11):e1010951. doi: 10.1371/journal.ppat.1010951 (PMC9674177; doi:10.1371/journal.ppat.1010951)

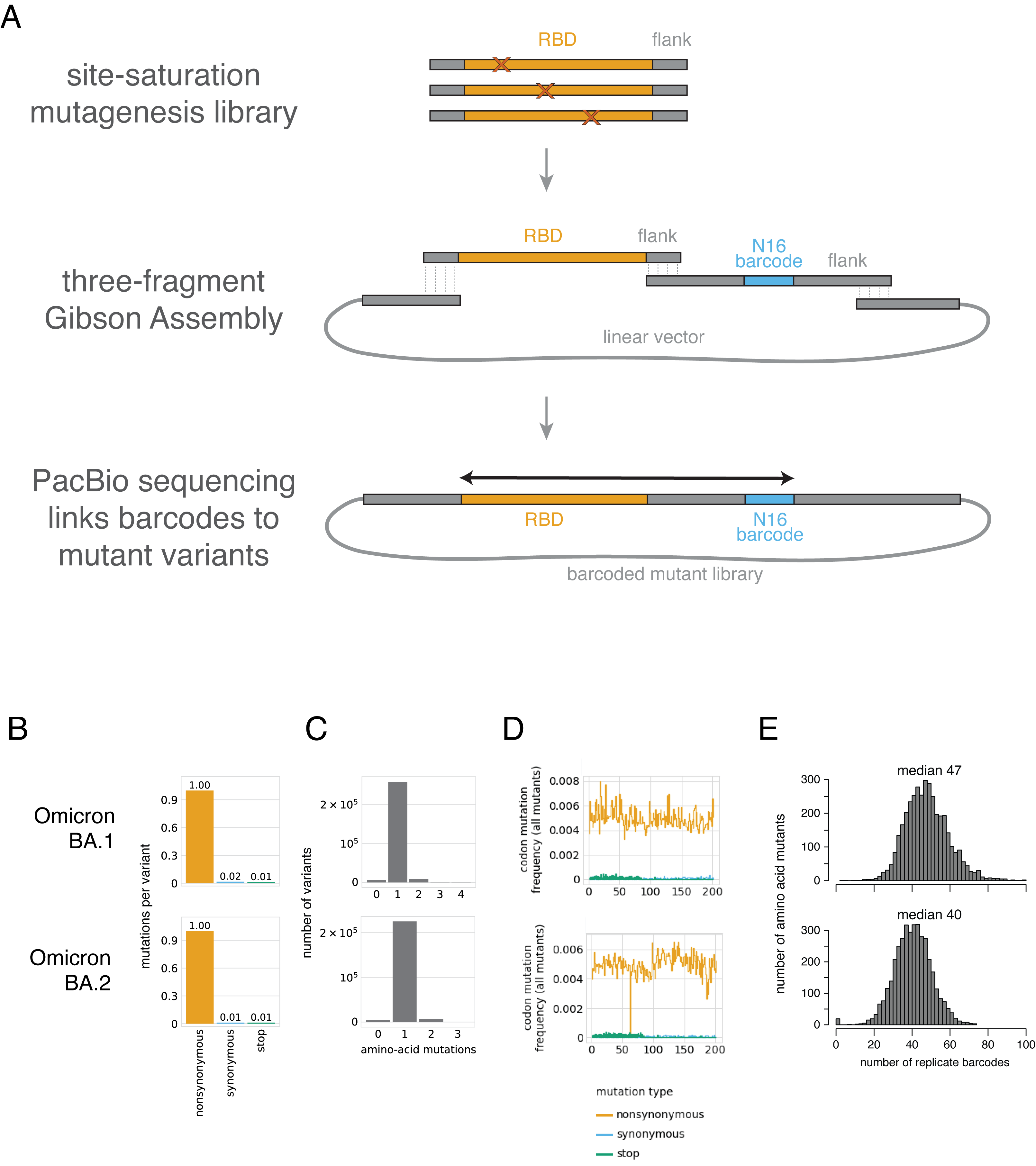

Supplement: S1 Fig — (A) Scheme for generation of the Omicron BA.1 and BA.2 RBD mutant libraries. Site saturation mutagenesis oligonucleotide libraries were constructed by Twist Bioscience with constant flank sequences. For each Omicron background, a three-fragment Gibson Assembly was performed with: (1) the pooled mutant RBD oligonucleotide, (2) a PCR-generated oligonucleotide encoding a randomized N16 nucleotide barcode, and (3) linearized vector backbone. PacBio sequencing of the barcoded mutant library plasmid was used to link N16 barcode to mutant RBD sequence, enabling complete definition of library statistics and the creation of a barcode-variant lookup table such that subsequent deep mutational scans only require N16 barcode sequencing. (B-E) For pooled duplicate Omicron BA.1 (top) and BA.2 (bottom) libraries, (B) the average number of mutations of each class per barcoded variant, (C) the distribution of the number of amino acid mutations per barcoded variant, (D) the mutation rate at each site along the RBD sequence, and (E) the distribution of the total number of associated N16 barcodes for each possible amino acid mutation (from filtered ACE2 binding scores). (TIF) [file ppat.1010951.s001.tif]

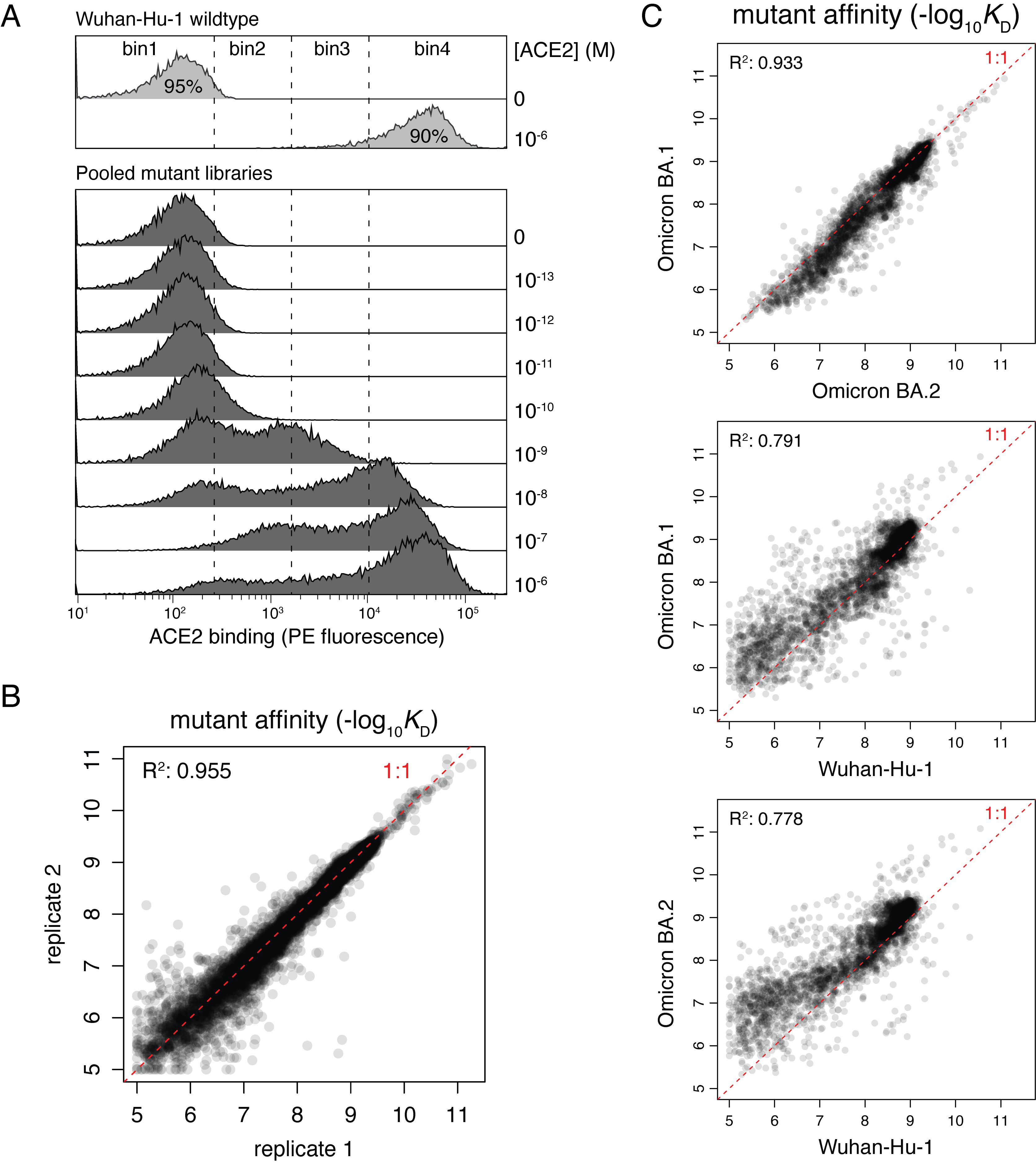

Supplement: S2 Fig — (A) Representative FACS scheme (replicate 1) used for ACE2-binding deep mutational scanning titration assays on pooled Wuhan-Hu-1, Omicron BA.1, and Omicron BA.2 mutant libraries. Bins of PE fluorescence (ACE2 binding) were drawn on cells pre-selected on FSC/SSC and FITC(RBD)/FSC plots to isolate single RBD+ cells. At each ACE2 concentration, 12.5 million cells total were collected across the four bins. Post-sort cells were sequenced to identify the distribution of counts of each library variant at each concentration, which were fit to titration curves to determine per-variant dissociation constants (KD). (B) Correlation in mutant ACE2-binding affinities in the pooled Wuhan-Hu-1, Omicron BA.1, and Omicron BA.2 libraries in experimental duplicates (independently barcoded and assayed mutant libraries). Red dashed line represents the 1:1 linear line. (C) Relationship between mutant ACE2-binding affinities in each of the RBD backgrounds that were assayed in parallel. Red dashed line represents the 1:1 linear line. Plots illustrate similar effects of mutations in the Omicron BA.1 and BA.2 backgrounds compared to the substantial variation in mutational effects in either Omicron background compared to Wuhan-Hu-1. (TIF) [file ppat.1010951.s002.tif]

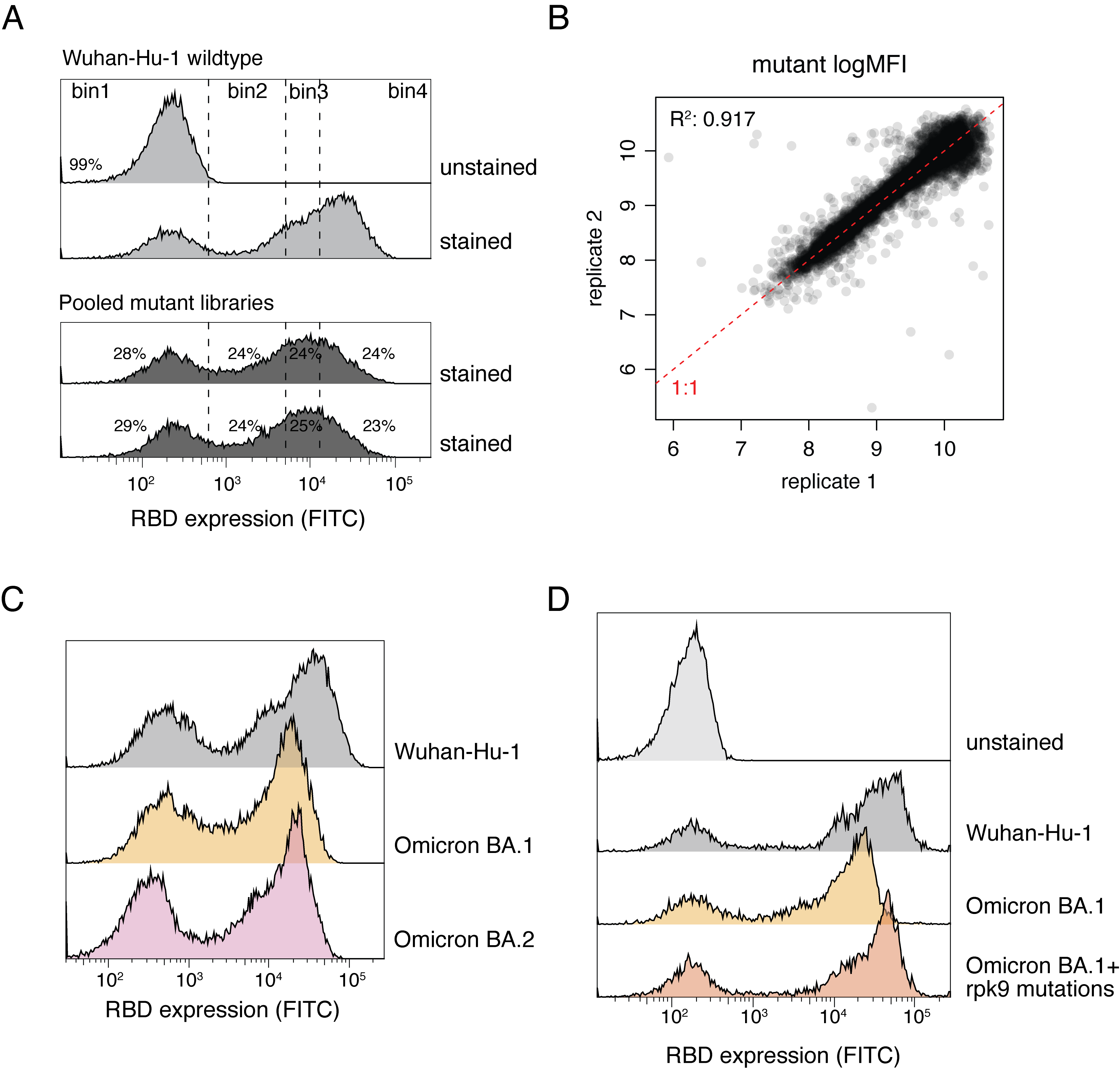

Supplement: S3 Fig — (A) FACS scheme used for RBD expression deep mutational scanning assays on pooled Wuhan-Hu-1, Omicron BA.1, and Omicron BA.2 mutant libraries. Bins of FITC fluorescence (RBD expression) were drawn on cells pre-selected on FSC/SSC plots to isolate single cells. Approximately 17 million viable cells were collected across the four bins and sequenced to identify the distribution of each library variant across bins, enabling the back-calculation of per-variant FITC mean fluorescence intensity (MFI). (B) Correlation in mutant RBD expression values in the pooled Wuhan-Hu-1, Omicron BA.1, and Omicron BA.2 libraries in experimental duplicates (independently barcoded and assayed mutant libraries). Red dashed line represents the 1:1 linear line. (C) RBD yeast-surface expression levels of Wuhan-Hu-1, Omicron BA.1, and Omicron BA.2 as measured by flow cytometry (D) RBD yeast-surface expression levels of Wuhan-Hu-1, Omicron BA.1, and Omicron BA.1 with the “rpk9” stabilizing mutations [27] as measured by flow cytometry. (TIF) [file ppat.1010951.s003.tif]

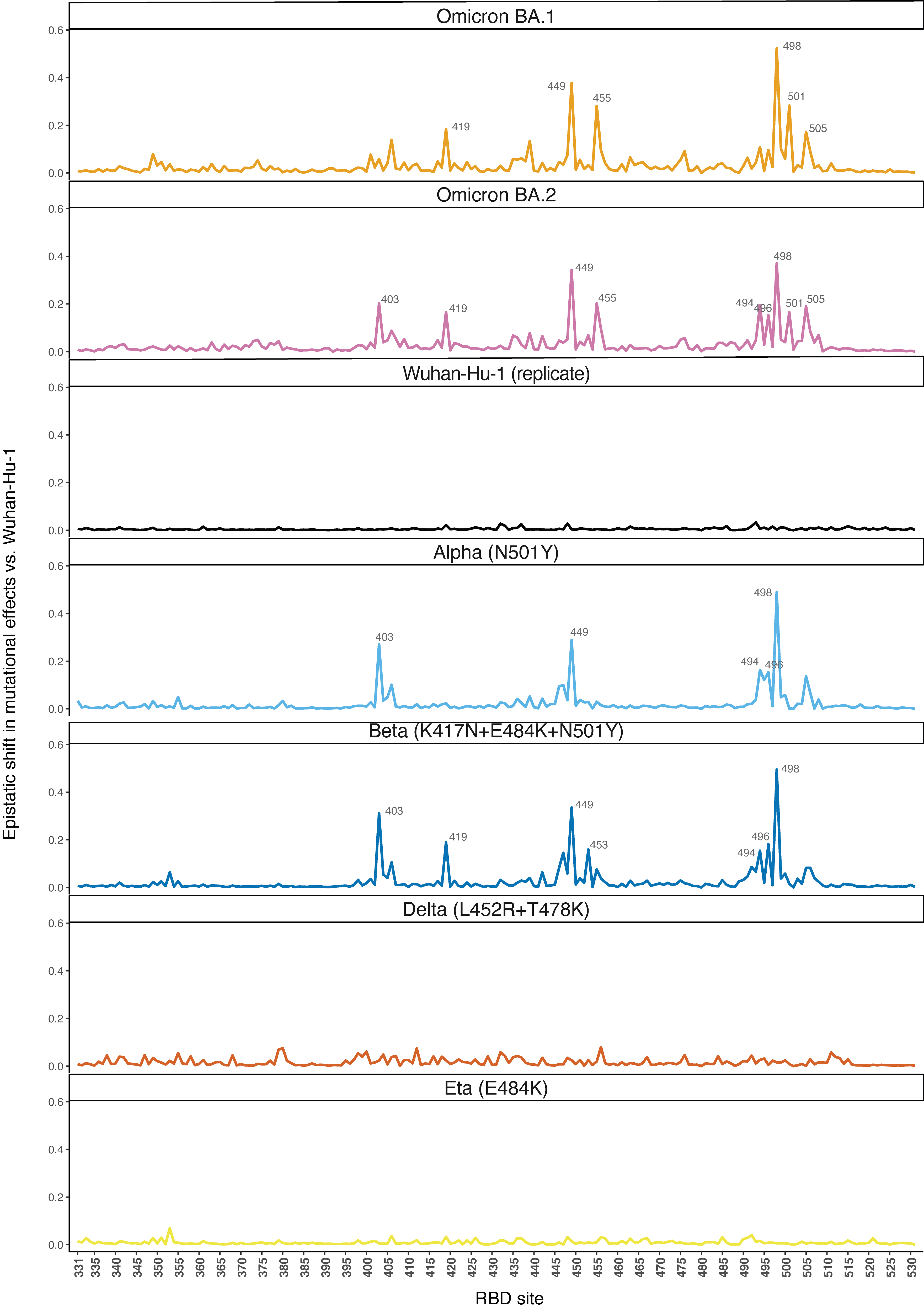

Supplement: S4 Fig — Faceted lineplots illustrate epistatic shifts in the effects of mutations on ACE2 binding at each RBD position compared to Wuhan-Hu-1 in all variant backgrounds for which we’ve conducted deep mutational scans. Interactive plot is available at https://jbloomlab.github.io/SARS-CoV-2-RBD_DMS_Omicron/epistatic-shifts/. All measurements are compared to the Wuhan-Hu-1 dataset generated in this study. Wuhan-Hu-1 (replicate) corresponds to our prior Wuhan-Hu-1 dataset that was gathered in parallel with the Alpha, Beta, and Eta datasets that are also represented here [10]. (TIF) [file ppat.1010951.s004.tif]

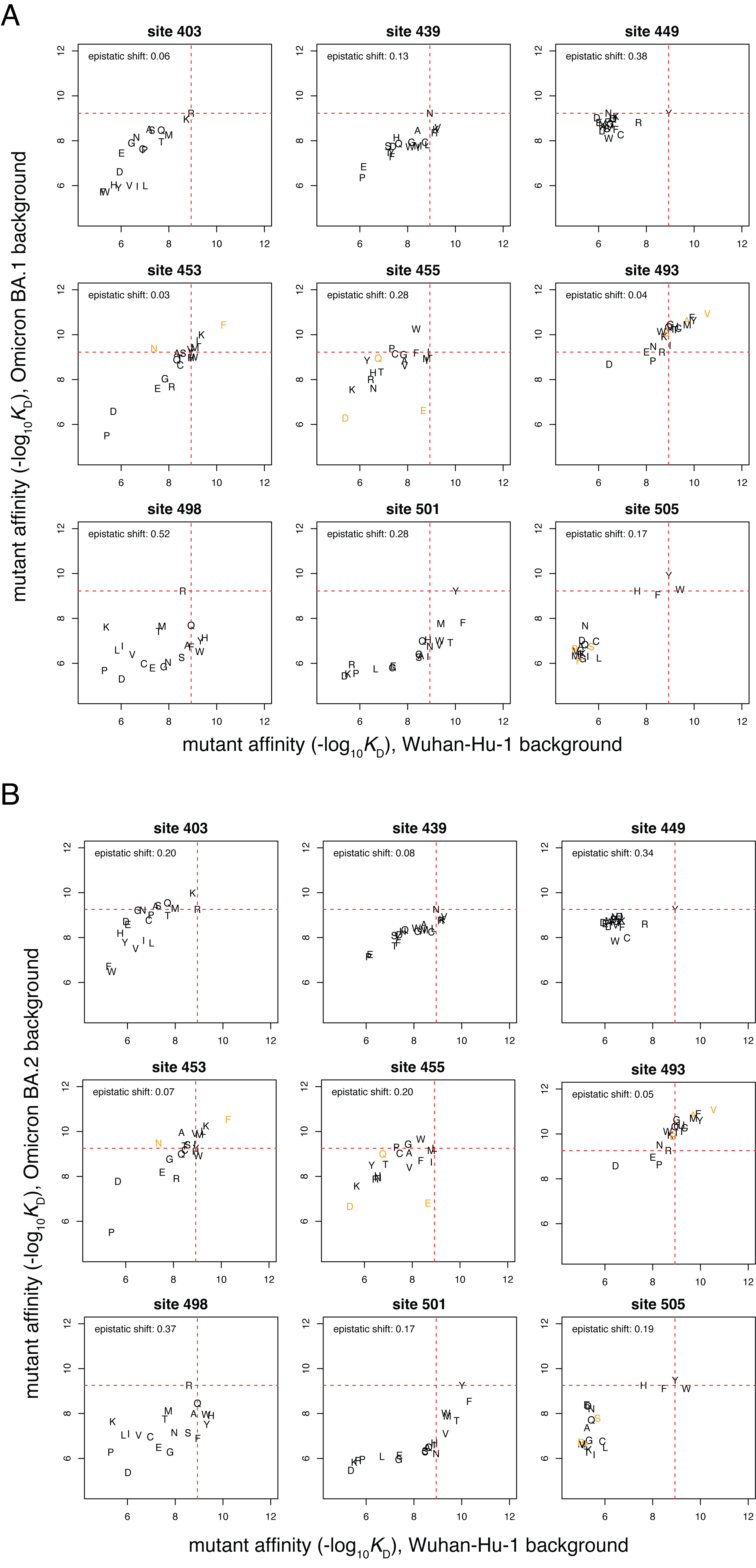

Supplement: S5 Fig — (A, B) Mutation-level plots of epistatic shifts at sites of interest. Each scatter plot shows the measured affinity of the 20 amino acids in the Omicron BA.1 (A) or BA.2 (B) versus Wuhan-Hu-1 RBDs. Red dashed lines indicate parental affinities. Orange letters are mutations that were sampled with fewer than 3 unique barcodes across titration replicates in one or both backgrounds and were not included in the epistatic shift computation. (TIF) [file ppat.1010951.s005.tif]

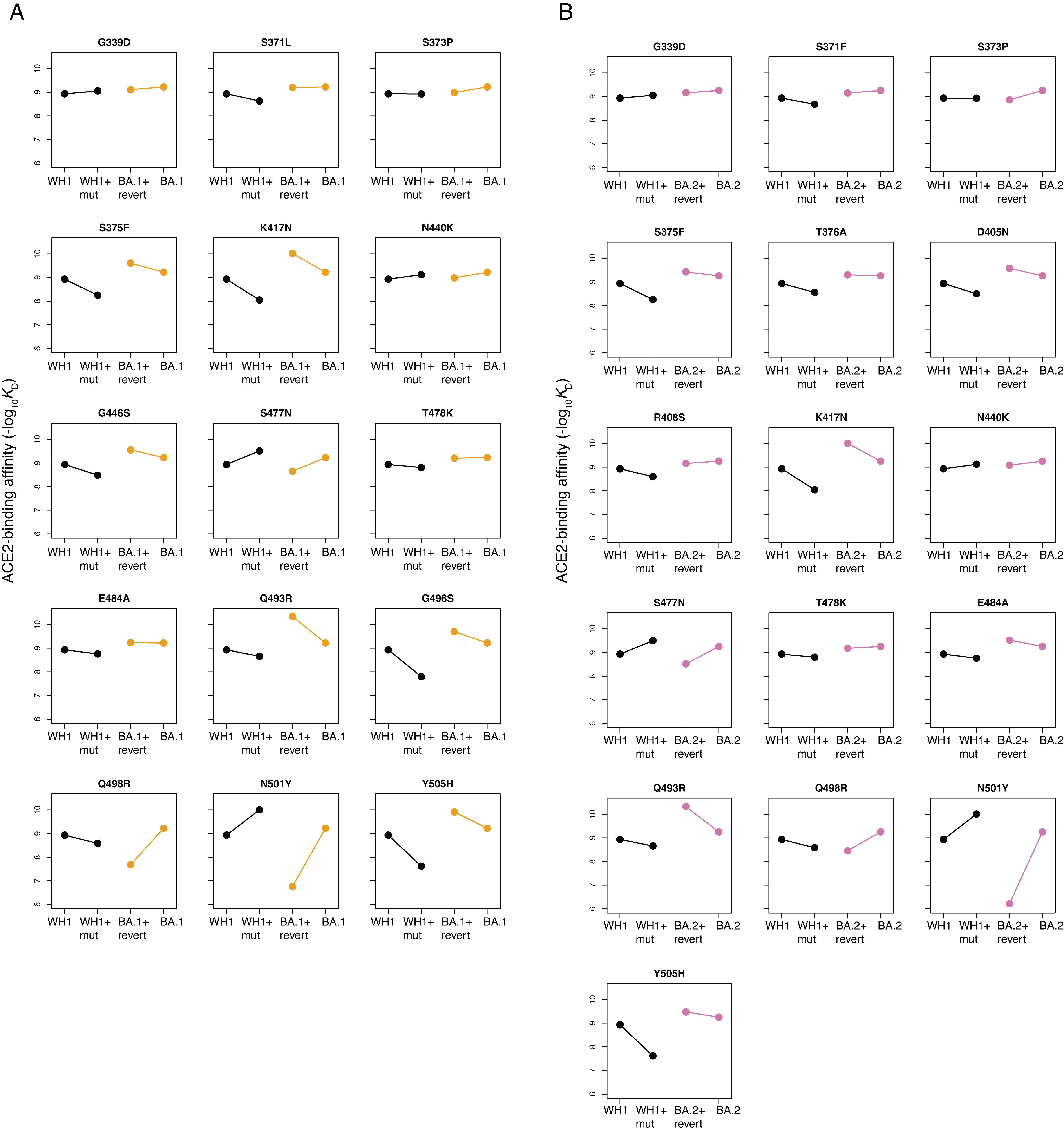

Supplement: S6 Fig — (A, B) For each substitution distinguishing Wuhan-Hu-1 from Omicron BA.1 (A) or BA.2 (B), the pattern of epistasis is reflected in the effect of the forward mutation in the Wuhan-Hu-1 background (WH1, black) versus its reversion in the Omicron (orange or pink) background. Entrenchment is evident in mutations where the slope of the orange or pink line is greater (more positive or less negative) than the slope of the black line (e.g., S371L, N501Y), and arises from favorable epistasis between the entrenched substitution and one or more other co-occurring substitutions. Mutations where the slopes are the same do not show epistasis with the other substituted Omicron positions (e.g., K417N). (TIF) [file ppat.1010951.s006.tif]

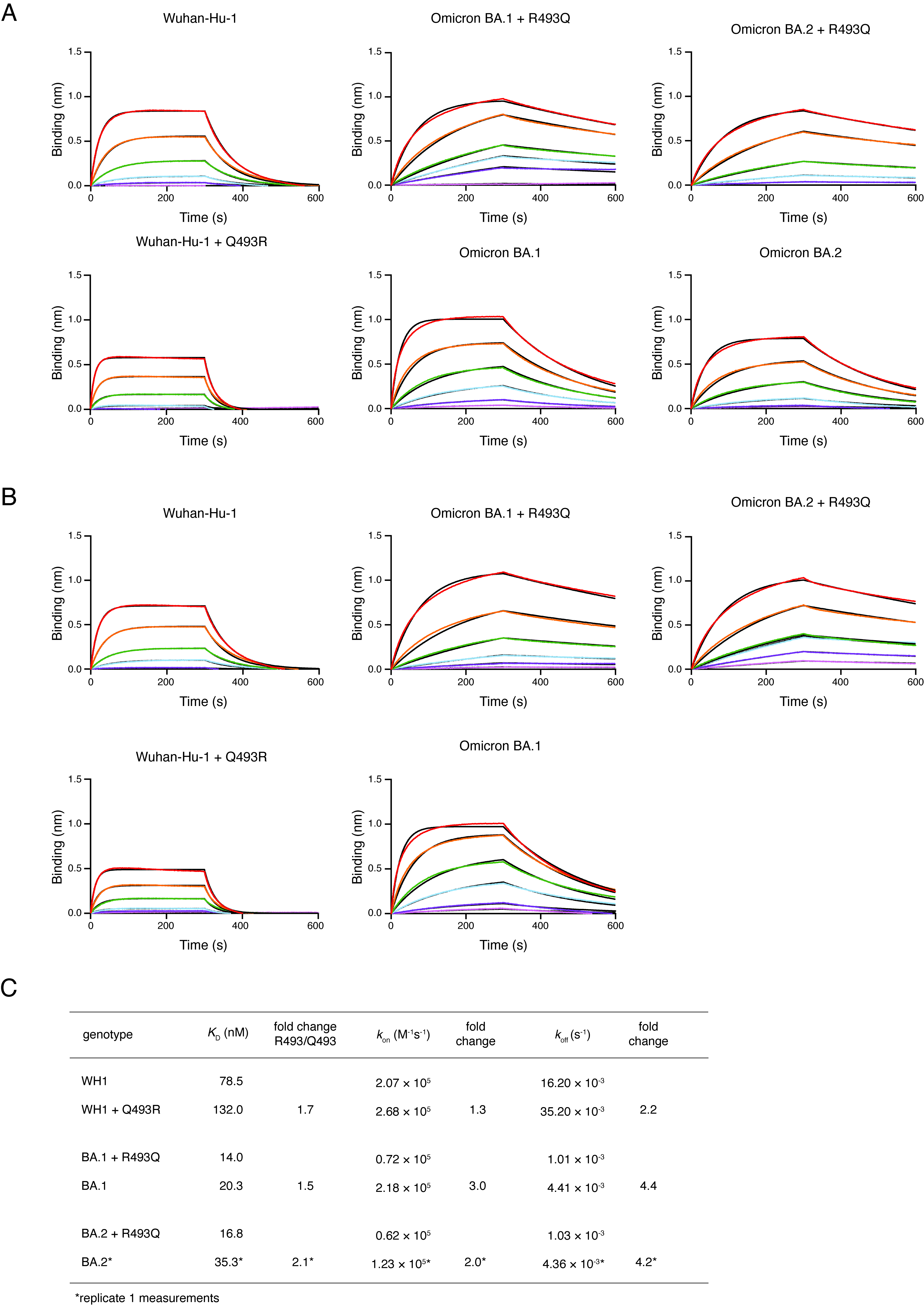

Supplement: S7 Fig — (A, B) BLI sensorgrams from experiments with RBDs immobilized on a BLI biosensor with monomeric human ACE2 as analyte. Binding curves were measured at ACE2 concentrations from 167 (red) to 0.7 (pink) nM at 3-fold dilutions, and kinetic parameters were inferred from a global fit. Curves in (A) represent the curves underlying the kinetic values given in Fig 3B, and curves in (B) represent a second replicate on independently purified RBD batches. (No second replicate for Omicron BA.2 was measured.) (C) Kinetic values for binding measurements shown in (B). Asterisk on BA.2 values indicates that these values are repeated from the replicate 1 experiments (A and Fig 3B). (TIF) [file ppat.1010951.s007.tif]

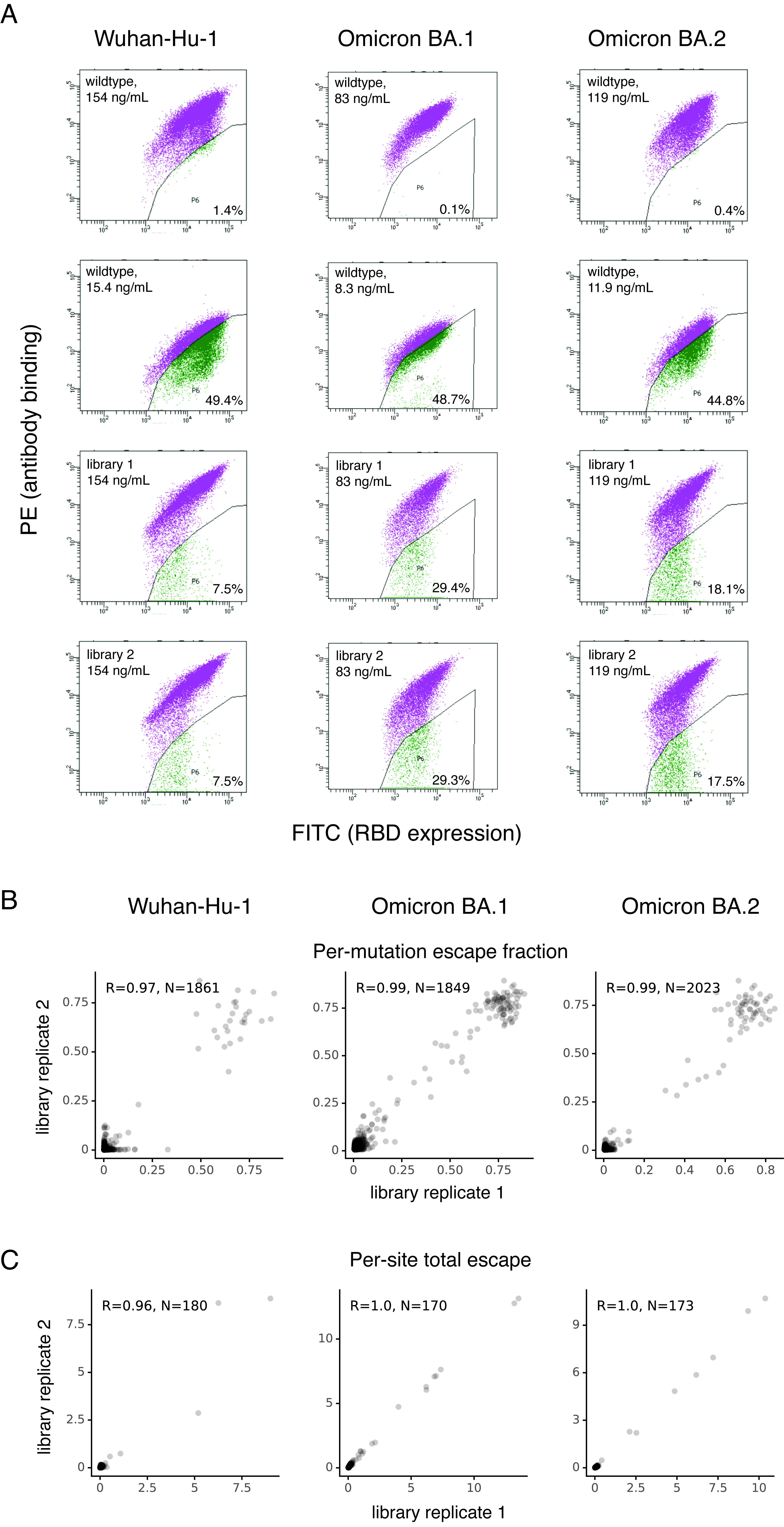

Supplement: S8 Fig — (A) FACS gates used to identify mutations that escape binding by LY-CoV1404 in each RBD background. For each experiment, an antibody-escape gate was drawn that captures approximately 50% of cells in the respective wildtype control labeled at 10% of the library selection antibody concentration. The “escape fraction” represents the fraction of cells of a mutant genotype that fall into the antibody-escape sort bin. (B, C) For each background, the correlation in the per-mutation escape fraction (B) or the sum of escape fractions of all mutations at a site (C). (TIF) [file ppat.1010951.s008.tif]
