# Supplementary material for: Deep mutational scans for ACE2 binding, RBD expression, and antibody escape in the SARS-CoV-2 Omicron BA.1 and BA.2 receptor-binding domains
Source: PLoS Pathog. 2022 Nov 18;18(11):e1010951. doi: 10.1371/journal.ppat.1010951 (PMC9674177; doi:10.1371/journal.ppat.1010951)
Supplement: Attachment — Submitted filename: response_to_reviewers.docx [file ppat.1010951.s012.docx]

Response to reviewers

Reviewer #1:

Starr et al. carried out deep mutational scans on SARS-CoV-2 Omicron BA.1 and BA.2 S RBD using their previously established workflow, as a continued effort in probing the RBD mutational landscape. They reported that, like previous variants, BA.1/BA. 2 are highly tolerant to mutation and can gain mutations that increase ACE2-binding affinity, yet some of those mutations were reversions to Wuhan-Hu-1. The authors also found reduced yeast surface expression of BA.1/BA.2 RBD compared to Wuhan-Hu-1 and listed potential stabilizing mutations within the Omicron RBD core. Epistatic shifts are also identified, including substitutions that show epistatic entrenchment. At the end, the author screened for LY-CoV1404 escape substitutions.

Overall, the experimental design and data presentation are straightforward and appropriate. While the results on epistatic shifts in this study are conceptually similar to one of the authors’ previous publications (PMID: 35762884), the finding on broadened pathways of escape from LY-CoV1404 is timely and corroborate with a recent preprint by another group (ref 41). The data in this study also provide a useful resource for the SARS-CoV-2 research field. I only have a few minor comments.

Minor Issues:

1.1. Lines 104-105: “We found that Omicron BA.1 and BA.2 RBDs have reduced yeast surface expression levels relative to Wuhan-Hu-1 (S2 Data and S3C Fig.)”. The data for BA.2 seem to be missing in Figure S3C?

Thank you for noticing this. The prior Fig. S3C shows flow cytometry experiments from a batch that did not include BA.2. We intended the S2 Data tag to give the complete quantitative comparison between Wuhan-Hu-1, BA.1, and BA.2, but we have added a new panel, Fig. S3C (moving the prior panel C to D), which shows matched flow cytometry data comparing the Wuhan-Hu-1, BA.1, and BA.2 yeast-display expression efficiencies.

1.2. Figure 1C: It seems like the mutation with no data available was labeled in yellow rather than the designated grey.

This was our mistake, we have updated the figure with the correct coloring for missing mutations.

1.3. Line 110: Should F492W be F392W instead?

That is correct, this typo has been fixed.

1.4. Lines 139-143: Is there a structural explanation on the distinctive effects of N439K and L455W on the ACE2 affinity between Wuhan-Hu-1 and Omicron RBDs, given that both ACE2-bound structures are available (PDB 6M0J and 7XO9)?

We did not see clear structural rationale for these different effects – across all three structures, these residues adopt identical conformations and are surrounded by similar amino acid residues in each structure (i.e., there’s not an obvious new contact that can be made by the mutations whose effects change across backgrounds). This is similar to what we saw when trying to rationalize epistatic shifts in the Beta RBD structure compared to Wuhan-Hu-1 in our prior study (citation 10).

Reviewer #2:

Previous work by the authors mapped the impact of single amino acid mutations in the receptor-binding domain (RBD) of SARS-CoV-2 on ACE2 binding and antibody escape. However, as the authors note, the impact of amino acid mutations in the RBDs of emerging SARS-CoV-2 variants may vary, due to differences in the genetic background of the novel virus subtype – a phenomenon known as epistasis. Thus, here the authors characterized the functional impact of single amino acid mutations in the RBD of the more recent Omicron BA.1 and BA.2 lineages of SARS-CoV-2 on binding to ACE2, and on escape from binding by a therapeutic monoclonal antibody (LY-CoV1404) using deep mutational scanning. Overall, the study is well-executed and compelling. Consistent with their hypothesis, the authors find the impact of some – but not all – amino acid substitutions on RBD function to differ in the Omicron lineages as compared to the ancestral Wuhan-Hu-1 strain, underscoring the importance of contextualizing predictions of SARS-CoV-2 protein function within the relevant genetic background. The authors further identify mutations in the Omicron BA.1 and BA.2 RBD likely to catalyze viral escape from the only clinically approved monoclonal antibody that potently neutralizes Omicron BA.1 and BA.2, which should inform SARS-CoV-2 genomic surveillance efforts and public health strategy as the Omicron lineages continue to evolve.

Major Issues:

2.1. In several locations in the manuscript (e.g. line 27), the authors claim to have determined the impacts of “all amino acid mutations” in the RBD. This can be considered misleading given that combinatorial mutations were not assessed, therefore it is more precise to say, “all single-site amino acid mutations”, or something similar.

This is a good point. We have updated this language on lines 27, 83, 124, and 233 to be more precise as the reviewer suggests.

2.2. Previous work by the authors used dimeric ACE2 (e.g. PMC7418704). What was their rationale for switching to monomeric ACE2 in this study? Do the results from DMS of the Wuhan-Hu-1 RBD remain consistent across the 2 ACE2 screening approaches?

We describe and included a plot illustrating the effects of mutations in Wuhan-Hu-1 on binding to dimeric versus monomeric ACE2 in our previous manuscript (PMID 35762884, citation 10). This shows consistent effects of mutations in the two approaches, but monomeric ACE2 better resolves mutational effects with small positive or negative effects on binding relative to wildtype, whereas dimeric ACE2 has less resolution near wildtype but extends the dynamic range to be able to distinguish large versus very large negative effects on ACE2 binding. We have added a sentence explicitly stating this rationale and this prior citation in line 90-91.

2.3. When describing Figure 1 in the text, several amino acid sites are alluded to. However, it is challenging to cross reference these sites in the figure with the relatively small heatmap tiles. Could sites of interest be highlighted more clearly in some way in the current figure. Alternatively, perhaps the heatmap could be expanded (and/or rotated 90 degrees) so the tiles are more clearly visible? Also, it is not immediately obvious what the yellow regions on the heatmaps in Figure 1 represent.

Apologies, the yellow in Fig. 1C was a mistake as corrected in response to point 1.2 above. We completely agree that the static heatmaps that can fit in a publication are challenging to directly reference. We therefore include a url to interactive heatmaps that are easier to visualize in the main text and in the Figure 1 legend. Elsewhere when we describe these specific sites, we refer to S5 and S6 Fig. which more helpfully shows the patterns at specific sites of interest, but we wish to wait to refer to S5 and S6 Fig. until later when we are describing more specific patterns instead of having the reader dive into that complexity early on when we are describing high level patterns.

2.4. The authors argue that the N501Y substitution shared between the Omicron and Beta RBDs is likely a “dominant determinant” that underlies the similar pattern of epistatic shifts in the Omicron and Beta variants. However, it is unclear how the authors arrived at this interpretation. A more robust discussion of this reasoning would be useful.

We were indeed being imprecise with this previous comparison. We previously saw that the epistatic shifts from Wuhan-Hu-1 to Alpha (N501Y) and Beta (K417N/E484K/N501Y) were very similar, and many of the sites perturbed in these two variants are also shifted in Omicron which shares N501Y. In contrast, in prior data on Delta (L452R/T478K) and Eta (E484K), we did not see these strong epistatic shifts. We have made this more explicit by modifying Fig. 2A and 2B (and associated text) to make the comparison with the Alpha variant (which contains N501Y alone) instead of the Beta variant (which has two additional RBD mutations on top of N501Y). This update does not alter our interpretations but now makes the focal comparison about N501Y much more precise and clear. We also added a new Supplemental Figure (S4 Fig.) illustrating the epistatic shift across all variants we have now scanned in this and our prior study (which are also all shown in the interactive visualization described in Fig. 2 legend), further emphasizing the dominant nature of N501Y in the epistatic shifts that we see across variants. Thank you for raising this and the following point, as these changes greatly clarify this section.

2.5. The authors suggest throughout the manuscript that the pattern of epistatic shift in the Omicron and Beta variants is similar. This broadly appears to be the case when examining Figure 2. However, this claim could be made stronger in a couple of ways. First, if their interpretation is correct, including another SARS-CoV-2 lineage as a benchmark in this analysis (e.g. Delta) would allow the authors to specifically show that the epistatic shift in mutational effects (vs. Wuhan-Hu-1) of Omicron are more similar to the Beta lineage when compared to other SARS-CoV-2 lineages; currently while Beta and Omicron visually look similar, there is no comparator. However, an additional DMS of the Delta variant RBD may be beyond the score of this paper. Thus, the authors might additionally consider using a quantitative, statistical metric to score similarity (or lack thereof) in epistatic shifts between variants (e.g. Beta and Omicron BA.1) in a pairwise manner to bolster their argument.

This is a great point. As described in point 2.4 above, we have added a new figure (S4 Fig.) that shows epistatic shifts from prior data on Delta (L452R/T478K) and Eta (E484K). These benchmarks help to emphasize the shared patterns of epistatic shifts in Omicron with other N501Y-containing variants.

Reviewer #3:

The authors built variant libraries of SARS-CoV-2 BA.1/BA.2 RBD using yeast surface display and performed deep mutational scanning assays to determine the impact on RBD expression and ACE2-binding affinity of each substition on BA.1/BA.2 RBD. They also analyzed the epistatic effects of Omicron's mutations and the escape profile of an important Omicron-neutralizing therapeutic NAb (LY-CoV1404). The data is trustworthy and helps a lot to explain the evolution of SARS-CoV-2 Omicron variants. These data has been widely accepted and cited by the community even before the submission of the manuscript. Therefore, I recommend this manuscript for publication despite two minor concerns to be addressed.

Minor issues:

3.1. Line 229. It's better to mention some designated names of newly emerging variants spreading rapidly that carry K444/V445 mutations and are highly likely to escape LY-CoV1404, such as BJ.1/XBB, BQ.1 and BR.1.

We have added more context to the sentence in lines 241-247 to highlight the vulnerabilities at residues K444 and V445 based on the citations we provide showing convergent evolution of these mutations in Omicron sub-variant evolution [39] and in wastewater surveillance [37, 38]. Because of the explosive proliferation of Omicron sub-variants (including those that are convergently sampling many mutations including K444/V445 as the reviewer points out), we will always be out of date with the newest lineage names du jour, so we choose not to mention specific lineage names for simplicity.

2. Fig. 4. Please use the ACE2-RBD complex structure with BA.1/BA.2 RBD (eg. PDB: 7WPB/7XB0) instead of Wuhan-Hu-1 RBD for projection of BA.1/BA.2-based DMS data.

We have appropriately updated the structural mapping for each background’s escape data to be on the corresponding variant ACE2-bound crystal structure in Figure 4.
